# Genomic diversity and population structure of teosinte (*Zea* spp.) and its conservation implications

**DOI:** 10.1371/journal.pone.0291944

**Published:** 2023-10-11

**Authors:** Diana María Rivera-Rodríguez, Alicia Mastretta-Yanes, Ana Wegier, Lino De la Cruz Larios, Fernando Santacruz-Ruvalcaba, José Ariel Ruiz Corral, Benjamín Hernández, José de Jesús Sánchez González

**Affiliations:** 1 Departamento de Ciencias Básicas, Tecnológico Nacional de México, Instituto Tecnológico de Tlajomulco, Tlajomulco de Zúñiga, Jalisco, México; 2 Centro Universitario de Ciencias Biológicas y Agropecuarias, Universidad de Guadalajara, Zapopan, Jalisco, Mexico; 3 Comisión Nacional para el Conocimiento y Uso de la Biodiversidad, Ciudad de México, Mexico; 4 Laboratorio de Genética de la Conservación, Jardín Botánico, Instituto de Biología, Universidad Nacional Autónoma de México, Mexico City, Mexico; KGUT: Graduate University of Advanced Technology, ISLAMIC REPUBLIC OF IRAN

## Abstract

The wild species of the genus *Zea* commonly named teosintes, comprise nine different taxa, distributed from northern Mexico to Costa Rica. Although this genus of plants has been extensively studied from a morphological, ecogeographical and genetic point of view, most contributions have been limited to the study of a few populations and taxa. To understand the great variability that exists between and within teosinte species, it is necessary to include the vast majority of known populations. In this context, the objective of this work was to evaluate the diversity and genomic structure of 276 teosinte populations. Molecular analyzes were performed with 3,604 plants and with data from 33,929 SNPs. The levels of genetic diversity by taxonomic group show a marked difference between species, races and sections, where the highest values of genomic diversity was found in ssp. *parviglumis* and ssp. *mexicana*. The lower values were obtained for the *Luxuriantes* section as well as ssp. *huehuetenagensi*s of the section *Zea*. The results of structure show that there is a great genetic differentiation in all the taxonomic groups considered. For ssp. *parviglumis* and *mexicana*, which are the taxa with the largest number of populations, a marked genomic differentiation was found that is consistent with their geographic distribution patterns. These results showed a loss of diversity in several teosinte populations, making a strong case for further collection, and *ex situ* and *in situ* conservation. Also, this study highlights the importance of integrating genomic diversity and structure for the applications of conservation and management.

## Introduction

Landscape transformation, mainly due to anthropogenic activities, has led to decreased diversity, size, and connectivity of many populations of plant species [1–5], including crop wild relatives (CWR) of Mesoamerica [5], which is one of the cradles of agriculture in the world [6]. Several Mesoamerican CWR can be naturally distributed in heterogeneous and fragmented landscapes [1, 2, 7], leading to genetic differences among populations. Observing changes in the evolutionary processes that originate and maintain CWR genetic diversity is key to conserving their adaptive potential in the face of climate change and human needs, both current and future [8]. Therefore, describing and monitoring the genetic diversity of Mesoamerican CWR is essential for their protection and management. For the case of maize (*Zea mays* ssp. *mays* L.), the Mexican Agreement on the Determination of Maize’s Centers of Origin and Diversity states that research should be carried on to characterize and monitor maize wild relatives’ genetic diversity at the population level [9]. Here, we contribute to fulfilling this task by focusing on the closest wild relatives of maize commonly known as teosintes, which comprise nine different taxa of the genus *Zea* (Poaceae).

**Fig 1:**
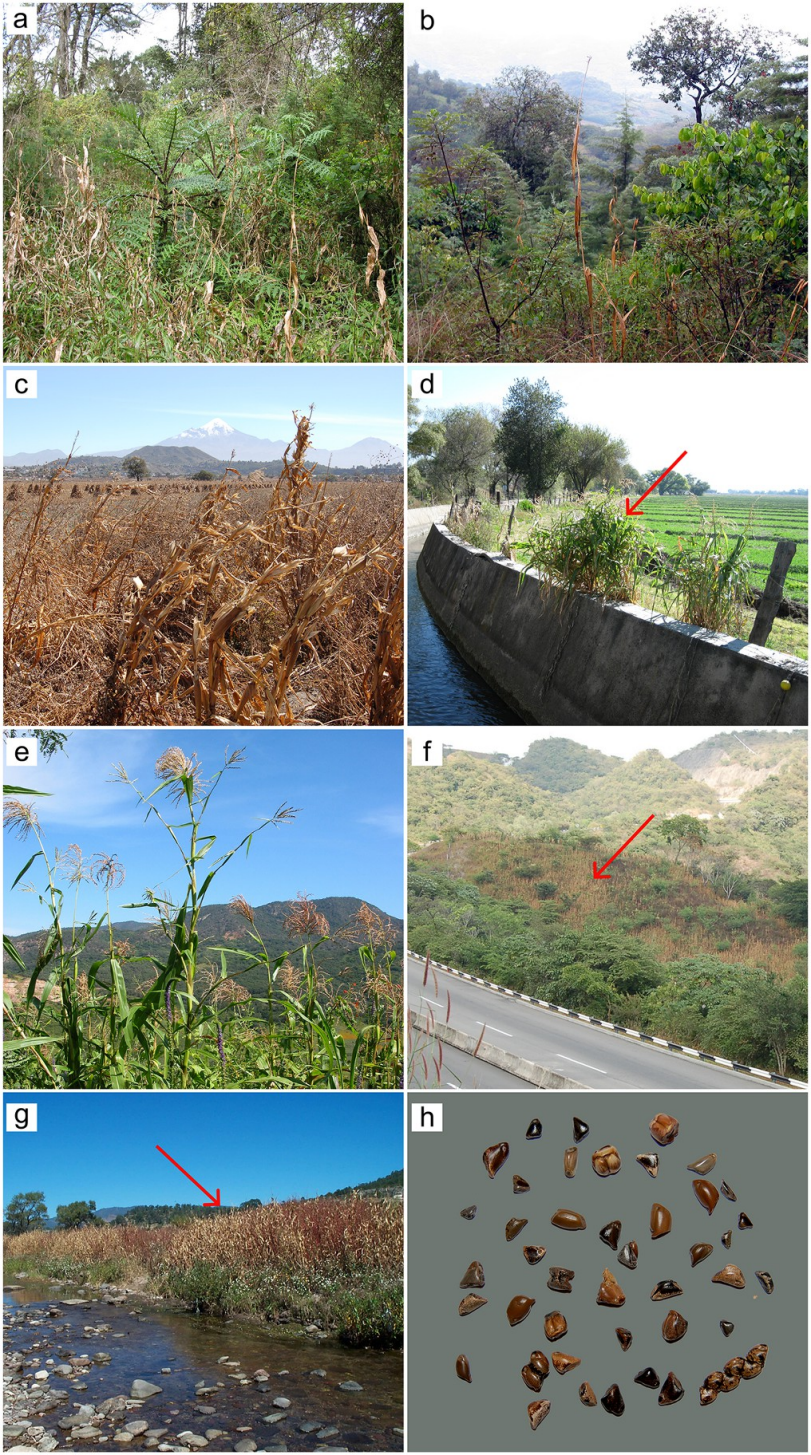
Variety of ecological conditions where teosinte populations can be found, ranging from the edges and within maize fields, on the edges of small streams, in open temperate or tropical forests, on rocky mountain slopes, and as a component of the herbaceous cover areas disturbed by anthropogenic activities. a) *Zea diploperennis* from Las Joyas, Cuautitlán de García Barragán, Jalisco, Mexico (2003); b) *Zea mays* spp. *huehuetenangensis* from Huehuetenango, Guatemala (2004); c) *Zea mays* ssp. *mexicana* race Chalco from Aljojuca, Puebla, Mexico (2007); d) *Zea mays* ssp. *mexicana* race Durango from Puente Dalila, Durango, Mexico (2009); e) *Zea mays* spp. *parviglumis* from Guachinango, Jalisco, Mexico (2003); f) *Zea mays* spp. *parviglumis* from Chilpancingo de los Bravo, Guerrero, Mexico (2005); g) *Zea mays* ssp. *mexicana* race Nobogame from Arrollo Tajamanil, Guadalupe y Calvo, Chihuahua, Mexico (2007); h) Seed diversity of teosintes. The red arrows highlight the teosinte plants. Photo credits: José de Jesús Sánchez González.

The genus *Zea* includes annual and perennial diploids (2n = 20) and a perennial tetraploid species (2n = 40) [10–12]. The study of the classification of teosintes starts with Wilkes [13], who identified geographic populations associated with different environments and described four races of teosinte for Mexico (Nobogame, Central Plateau, Chalco and Balsas) and two for Guatemala (Guatemala and Huehuetenango). Today, the genus is divided into two sections based on the morphological, ecological, and molecular features [10–12]: Section *Luxuriantes* and section *Zea*. Section *Luxuriantes* includes: perennial diploid species *Zea diploperennis*, perennial tetraploid *Z*. *perennis*, and the annuals diploids *Z*. *luxurians*, *Z*. *nicaraguensis* and *Z*. *vespertilio*. *Z*. *diploperennis* is distributed in very small populations exclusively in: (i) a small valley in the mountains of the Sierra Madre Occidental, in the locality of San Andrés Milpillas at Huajicori, Nayarit at an average altitude of 1,400 meters above sea level (m asl), (ii) at the slopes of Cerro de San Miguel, inside of natural protected area of Las Joyas at Sierra de Manantlán, municipality of Cuautitlán, Jalisco, at altitudes from 1,400 to 2,400 m asl. *Z*. *perennis* populations are restricted to El Fresno locality at Uruapan, Michoacán, at an average altitude of 1,380 m asl; and on the slopes of Volcán de Colima in the state of Jalisco at altitudes of 1,600–2,200 m asl. *Zea luxurians* is native to southeastern Guatemala, Honduras, El Salvador and southern México at altitudes between sea level and 1,100 m asl. Subsequently, *Z*. *nicaraguensis* was described by Iltis and Benz [14] as a geographically isolated annual teosinte from the coastal plain and estuaries near the Gulf of Fonseca, Nicaragua at elevations of 9 to 75 m asl; most populations are small and restricted to the Department of Chinandega at Rancho Apacunca, Cayanlipe and El Rodeo. Finally, *Z*. *vespertilio* was recently described at Murciélago Islands, Santa Elena Península, Guanacaste province of Costa Rica [15]. The Section *Zea* includes only three subspecies of *Zea mays*: *Zea mays* ssp. *huehuetenangensis* (to which we refer to as ssp. *huehuetenangensis* from now on), which is only found in western Guatemala at elevations of 900–1,650 m asl at San Antonio Huista, Jacaltenango, Santa Ana Huista and Nenton localities; and *Zea mays* ssp. *parviglumis* and *Zea mays* ssp. *mexicana* (ssp. *parviglumis* and ssp. *mexicana*, respectively, from now on) which occupy a diverse geographic range in Mexico, showing some level of geographic overlap within their natural distribution in central and northern Mexico. Subspecies *mexicana*, is distributed among highlands of the Mexican High Plateau and Sierra Madre Occidental biogeographical provinces, and ssp. *parvilgumis*, is found among the Mexican southwest lowlands of the Balsas basin and Sierra Madre del Sur province [16]. Efforts of phenotypic and ecogeographic characterization clearly separate ssp. *mexicana* into four races [17, 18]: race Chalco is found in the Toluca Valley and Chalco-Amecameca-Texcoco, Mexico State and Ciudad Serdán and Puebla in Puebla State. The Central Plateau race occurs in the states of Guanajuato, Michoacán, and Jalisco. Race Nobogame is restricted to southern Chihuahua. Race Durango is found near the city of Durango and in the county of Nombre de Dios, Durango. All together, teosintes encompass a wide range of environmental conditions and habitats, ranging from sea level to more than 2,000 m asl, and well as both areas with conserved natural vegetation, maize fields and sites with anthropogenic disturbances (Fig 1).

Teosintes are relevant for agriculture because their genetic variation may be useful in maize breeding programs, for example: aerenchyma in the roots for adaptations to rains and floods of *Z*. *luxurians* and *Z*. *nicaraguensis* [19, 20]; virus-resistant *Z*. *perennis* and *Z*. *diploperennis* [21]; resistance to parasitic plants such as *Striga* spp. from *Z*. *diploperennis* [22]; and very short vegetative growth of the *Z*. *mays* ssp. *mexicana* Durango race, which allows it to survive extreme drought [16]. Additionally, teosintes are associated with the culture of many local communities of México and Central America, where they are used to feed livestock, make traditional handcrafts and even as medicine [23], and noteworthy in some areas farmers keep teosinte plants in their fields, because they believe teosinte can help maize to better tolerate drought and pests [24]. Today, these cultural and applied uses are threatened by the transformation of the environmental context where teosintes’ genetic diversity has been maintained under constant evolution [12, 16, 25].

Evolutionary scientific interest on teosintes has mostly focused on systematics [12], showing results congruent with morphological data and the taxonomic recognition of subspecies [26, 27], describing new teosintes [28], or even understanding how the domestication of maize occurred from its two closest wild relatives (ssp. *parviglumis* and ssp. *mexicana*) [29, 30]. The most common ultimate goal in teosinte research is to identify the genetic basis of desirable characteristics for maize breeding, but only few studies have focused on populations as units where evolutionary processes occur. For example, van Heerwaarden et al. [31] determined the genetic structure within ssp. *parviglumis*, confirming a high level of gene flow among populations and a low, but clear level of genetic differentiation, due to geographic features. Hufford et al. [32] suggested that an introgression between ssp. *mexicana* and maize occurred during the expansion of the crop into the highlands of central Mexico early in its domestication process, leading to the incorporation of adaptive alleles of ssp. *mexicana* into the early maize. Focusing on both ssp. *mexicana* and ssp. *parviglumis*, Pyhäjärvi et al. [33] found that genetic structure follows complex patterns, created by altitude, dispersal events, and admixture among subspecies. In those same subspecies, Aguirre-Liguori et al. [34] found that divergence has occurred or been maintained despite geneflow, with putative inversions contributing to reduced gene flow between locally adapted populations. In summary, previous studies have shown that genetic diversity within teosinte taxa is structured and subjected to processes of local adaptation and divergence despite a history of past and modern gene flow. Untangling this structure is of conservation concern, because to truly represent the genetic diversity of teosintes it is necessary to focus conservation efforts into genetically distinct populations. However, previous studies have focused on few taxa (mostly only ssp. *mexicana* and ssp. *parviglumis*), few sampling localities within taxa, and few samples per sampling locality [26–29, 31–34]. In order to guide the effective management and conservation planning of teosintes including the genetic level, here we evaluate the diversity and genomic structure of all the wild taxa of the genus *Zea* (except *Zea vespertilio*) using SNP markers from 276 sampling localities and 3,604 individuals.

## Materials and methods

### Plant material and DNA extraction

**Fig 2:**
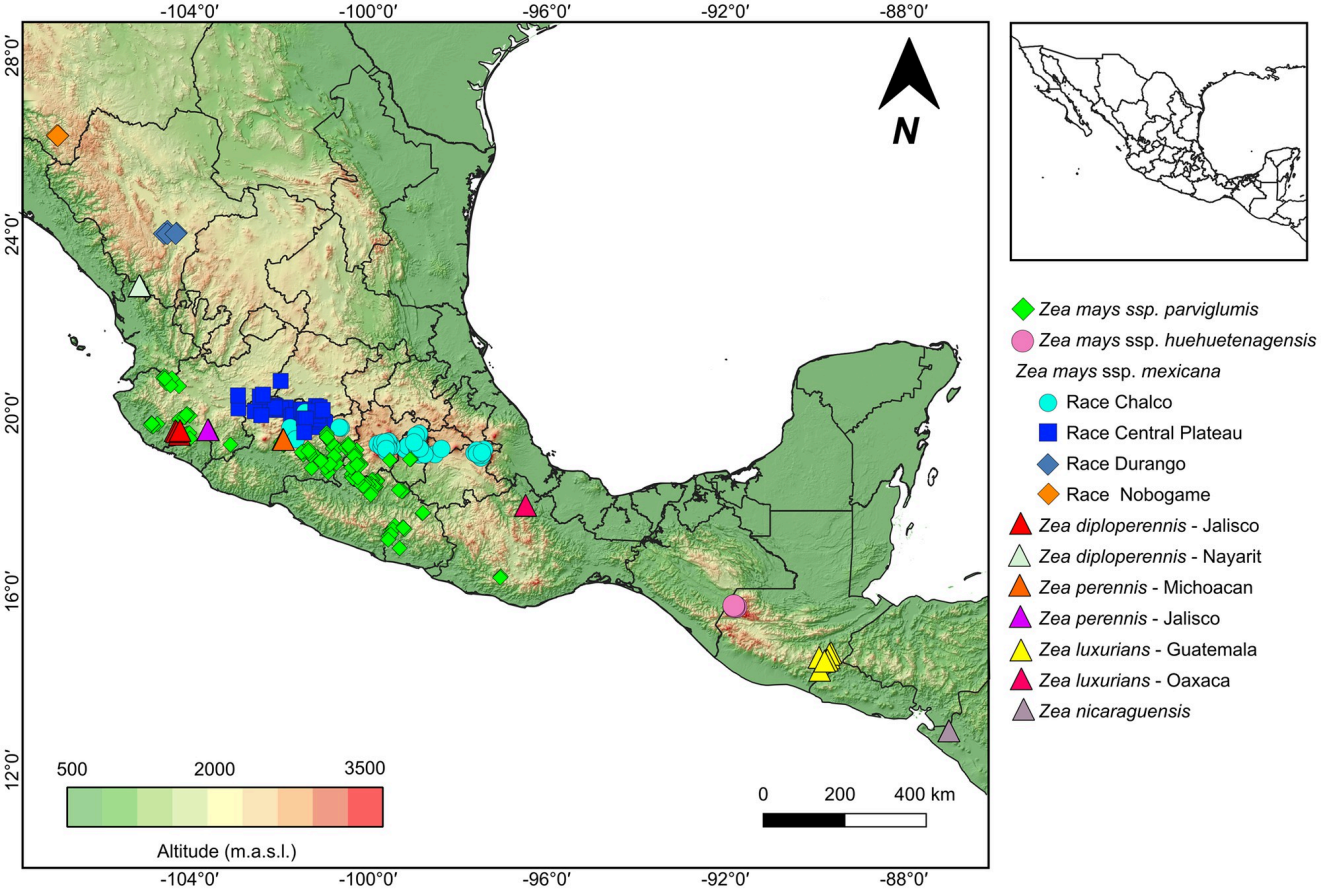
Geographic distribution of the 276 populations of seven teosinte taxa used for this study. Topographic background of Digital Elevation Model from Worldclim [37]. Resolution 1km.

Plant material for this study was obtained from 276 teosinte populations representing each of the known *Zea* species and subspecies (except *Zea vespertilio*, which was recently described [15] and for which no seed samples were available for the present study) and their races, throughout their entire geographical distribution from northern Mexico to western Nicaragua (Fig 2) (S1 Data). The accessions were provided by Instituto de Manejo y Aprovechamiento de los Recursos Fitogenétios (IMAREFI) of the Centro Universitario de Ciencias Biológicas y Agropecuarias (CUCBA) of the Universidad de Guadalajara, Jalisco, Mexico and International Maize and Wheat Improvement Center (CIMMYT). Most (182 out of 276) population were sampled between 2007–2010 as part of CONABIO’s Global Maize Project [35], few were from the 1960–90’s (9) or after 2010 (3), and the rest from the early 2000’s (82) (S1 Data). The number of individual plants per population was 30 for the 20 type populations and of 15 for the rest (256 populations). Plants were grown from seeds in greenhouse conditions at CUCBA, Jalisco, Mexico during 2014 and 2015. The work of molecular biology was carry out by the Laboratorio de Genética de la Conservación at Jardín Botánico of Instituto de Biología, Universidad Nacional Autónoma de México (UNAM). DNA was obtained from mainly young leaves a 2xCTAB with PVP-40 protocol [36] DNA quality was assessed in agarose gels.

### Sequencing

Library preparation and sequencing for Genotyping-By-Sequencing (GBS) was performed at the Institute for Genomic Diversity (Cornell University, Ithaca, NY, USA) following a GBS protocol [38]. DNA was digested with the ApeKI methylation-sensitive 5 base-pair (bp) recognition site restriction enzyme. The resulting fragments were ligated to Illumina HiSeq 2500 sequencing adapters and to adapters with sequence barcodes unique to each individual sample. GBS libraries were made in 96-sample plates (96-plex with 95 samples and one empty random cell). In total, 48 sequenced libraries were obtained, each with an average size of 15 GB output data. A total of 4,010 individuals were sequenced, including few duplicated samples for quality comparison and were discarded from downstream analyses.

### SNP calling and filtering

The sequence data and the genotypic database of SNPs were processed in the Tassel-5-GBS Production Pipeline software [39]. Using as reference draft ZeaGBSv2.7 Production (TOPM Tags On Physical Map); which contains genotypes from a collection of more than 60,000 maize samples. A total of 955,690 SNPs distributed throughout the genome were called, of which 955,120 mapped to chromosomes 1–10, and 570 did not map to any chromosome. This first SNPs database (Teo_phase_1_ZeaGBSv27raw) was subsequently filtered in Tassel by: (1) number of reads (Set Low Depth Genos to Missing, with a minimum value of 2); (2) frequency of the minor allele of at least 5% (MAF> 0.05) and; (3) loci present in at least 60% of the individuals. The resulting data was of 136,212 SNPs, which went to another filtering stage with Plink 1.9 [40], using the following criteria: keep only SNPs under linkage equilibrium and loci present in at least 80% of the individuals (–indep-pairwise 50 10 0.2—gene 0.2). Quality control for teosinte individuals excluded duplicated individuals and individuals with the highest missing data. The final database used for downstream analyses included 33,929 SNPs of 3,604 teosinte plants. Single nucleotide polymorphism (SNP) data used for the genomic diversity and population structure of teosinte are available at Dryad Data Repository (https://doi.org/10.5061/dryad.2547d7wxp).

### Data analysis and genetic diversity

We estimated genomic diversity indices in two ways: the first included independent estimates for each of the 276 populations, while the second grouped the 276 populations according to the taxonomic classification criterion including the levels to subspecies and races (13 taxa). We calculated the observed heterozygosity (*Ho*) and expected heterozygosity (*He*) with the 4P v. 1.0 software [41]. To identify taxa and their teosinte populations with very low, low, medium, and high *Ho* and *He* index, we partitioned the values into percentiles and quartiles and identified the species that fall in the lowest and highest percentile based on four thresholds: 1) <10th percentile; 2) 10th percentile to 2nd quartile; 3) from the 2nd quartile to the 90th percentile and 4) >90th percentile. This classification criterion was made based on the proposal of Canteri et al. [42] with modifications. Finally, they were represented by their geographical distribution.

Nucleotide diversity (π), Polymorphic site (PS), Theta of Watterson (θw) and Heterozygosity sites (*Hs*) were obtained with DnaSP [43]. Each of the diversity measures was visualized using boxplots with R [44]. Isolation by distance (IBD) was evaluated using a Mantel test with the use of a pairwise matrix distance of the fixation index (F_*ST*_) (S2 Data) and the geographical distance (S3 Data). This analysis was performed only for the spp. *parviglumis*, spp. *mexicana*, *Z*. *luxurians* and *Z*. *diploperennis* because they are the taxa with enough data points to perform the test. The F_*ST*_ matrix was estimated in Arlequin 3.5 [45]. For the IBD analysis, the *adegenet* package [46] of R was used.

### Population structure

We examined population structure to identify clusters of genetically related individuals within all teosinte taxa. For this, we: (1) generated a pairwise genetic matrix according to the Tamura-Nei distance using Mega 7 software [47]; (2) performed a principal coordinate analysis (PCoA) using the option Dcenter, Eigen and Mod3D of NTSYS 2.21s software [48]; (3) computed an analysis of molecular variance (AMOVA) of the SNP data using Arlequin to examine the partitioning of genetic variability among and within population and taxa; (4) performed a Discriminant Analysis Principal Components (DAPC) to identify and describe clusters of genetically related individuals among all taxa together. DAPC was carried out using the optimal number of groups (K), which was determined based on the K-means algorithm that uses the Bayesian Information Criterion (BIC). BIC values were calculated for K 1 to 40 and 3,300 PCs retained. DAPC was carried out with the *adegenet* package from R. Based on the best K group from the DAPC analysis, an AMOVA was performed to determine the differences between and within the assigned groups with Arlequin. And (5) additionally, to examine the level of admixture among individuals and between populations with higher detail, for ssp. *parviglumis* and ssp. *mexicana*, we run the software Admixture [49] with K = 1 to 60 and K = 1 to 50, for each taxon respectively. The CV error of all K was compared and the K value where the slope changed was used for plotting. We focused on only those taxa because they are two ones more widely distributed showing a more complex population structure in the DAPC.

## Results

A total of 276 populations of nine taxa were sequenced and genotyped using GBS. A total of 4,010 teosinte plants were sequenced, of which 80% met the molecular quality and filtering parameters. The final matrix used for analyses included 33,929 SNPs and 3,604 individuals.

### Genomic diversity

**Fig 3:**
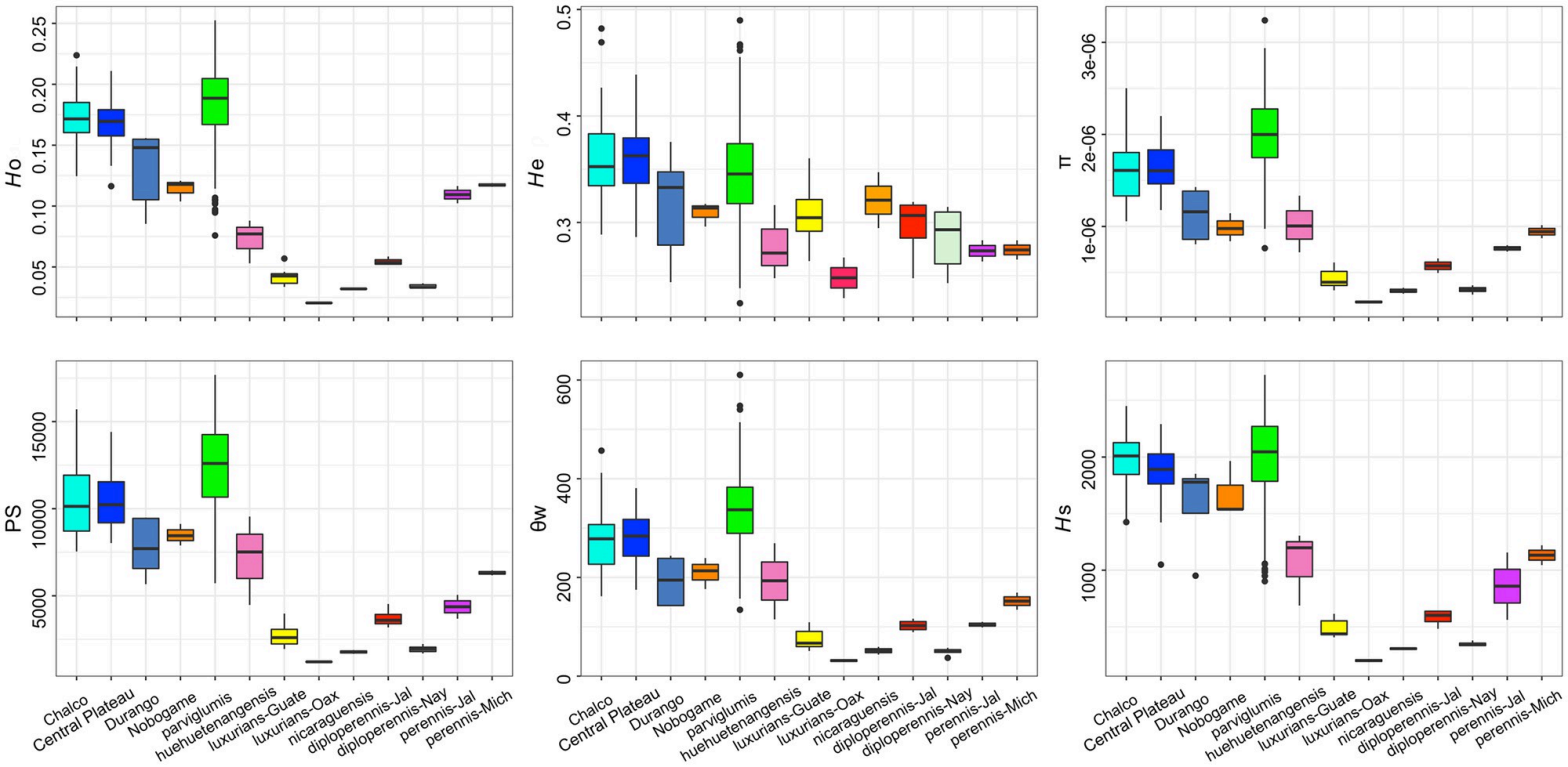
Genetic diversity indexes of 13 teosinte taxa. *Ho*: Observed heterozygosity; *He*: Expected heterozygosity; π: nucleotide diversity, PS: Polymorphic sites; θw: Theta of Watterson; *Hs*: Heterozygosity sites. Colors match Fig 2.

**Fig 4:**
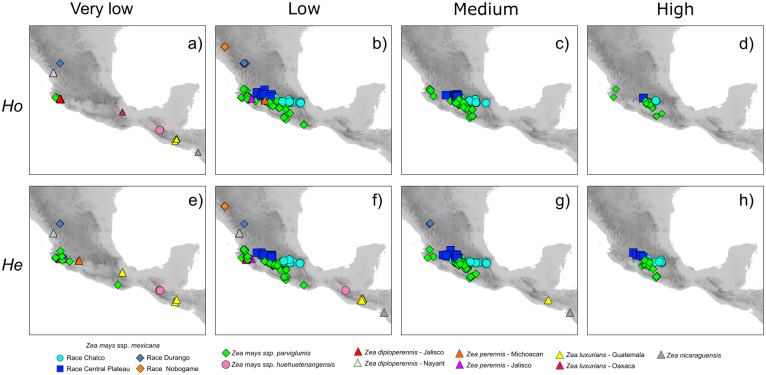
Observed heterozygosity (*Ho*) and expected heterozygosity (*He*) values of 276 teosinte populations based in 33,929 SNPs. The panels indicate the population frequency divided in very low (a and e), low (b and f), medium (c and g) and high (d and h) values respectively for each index. For *Ho*: Very low = <0.0965, Low = 0.0965 to 0.1726, Medium = 0.1726 to 0.2104 and High = >0.2104. For *He*: Very low = <0.2833, Low = 0.2833 to 0.3449, Medium = 0.3449 to 0.4038 and High = >0.4038.

**Fig 5:**
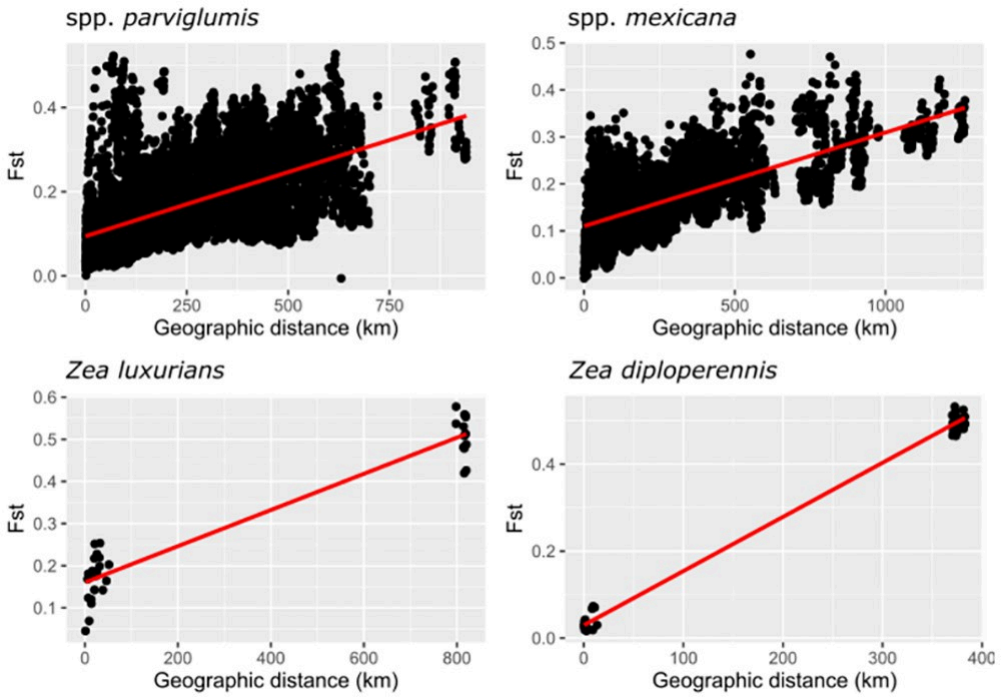
Isolation by distance model for ssp. *parviglumis* (142 populations, R^2^ = 0.32, obs = 0.59), ssp. *mexicana* (104 populations, R^2^ = 0.48, obs = 0.69), *Z*. *luxurians* (9 populations, R^2^ = 0.92, obs = 0.65) and *Z*. *diploperennis* (10 populations, R^2^ = 0.99, obs = 0.99). All tests were significative (*p-value* 0.001).

The values of genetic diversity parameters based on the 33,929 SNPs examined for the 276 teosinte populations are presented in S4 Data. The levels of genetic diversity by taxonomic group show a marked difference between species, races and sections (Fig 3). The diversity parameters of π, PS, θw and *H*s presented the highest values of genomic diversity in ssp. *parviglumis*, followed by ssp. *mexicana* but with variation between races. Lower values were obtained for the *Luxuriantes* section as well as ssp. *huehuetenangensis* of the section *Zea*. For the taxa of the *Zea* section, the values of *H*o are variable, with ssp. *parviglumis* showing the highest values but with a wide range among populations. For races of ssp. *mexicana*, the highest values were found within races Chalco and Central Plateau (Fig 3). *H*e values were higher than *H*o, however they show the same patterns. Within population values were variable depending on the taxonomic group (Fig 3). The spatial representation of the *H*o indicates that the populations with the lowest values are those with small population sizes and which are geographically isolated in northern Mexico or Central America; they include most of the populations of section *Luxuriantes* and races Nobogame and Durango (Fig 4a). The populations of more widely distributed taxa showed intermediate values, and most of them distributed in western and central Mexico (Fig 4b). The spatial representation of the lowest values of *He* shows a wider distribution than for *H*o (Fig 4d); the same is true for the populations with intermediate values (Fig 4e). The populations with the highest values of *He* are distributed in central Mexico but with less frequency than for *Ho* (Fig 4f). Lastly, all four analyzed taxa showed positive IBD (Fig 5).

### Population structure

**Fig 6:**
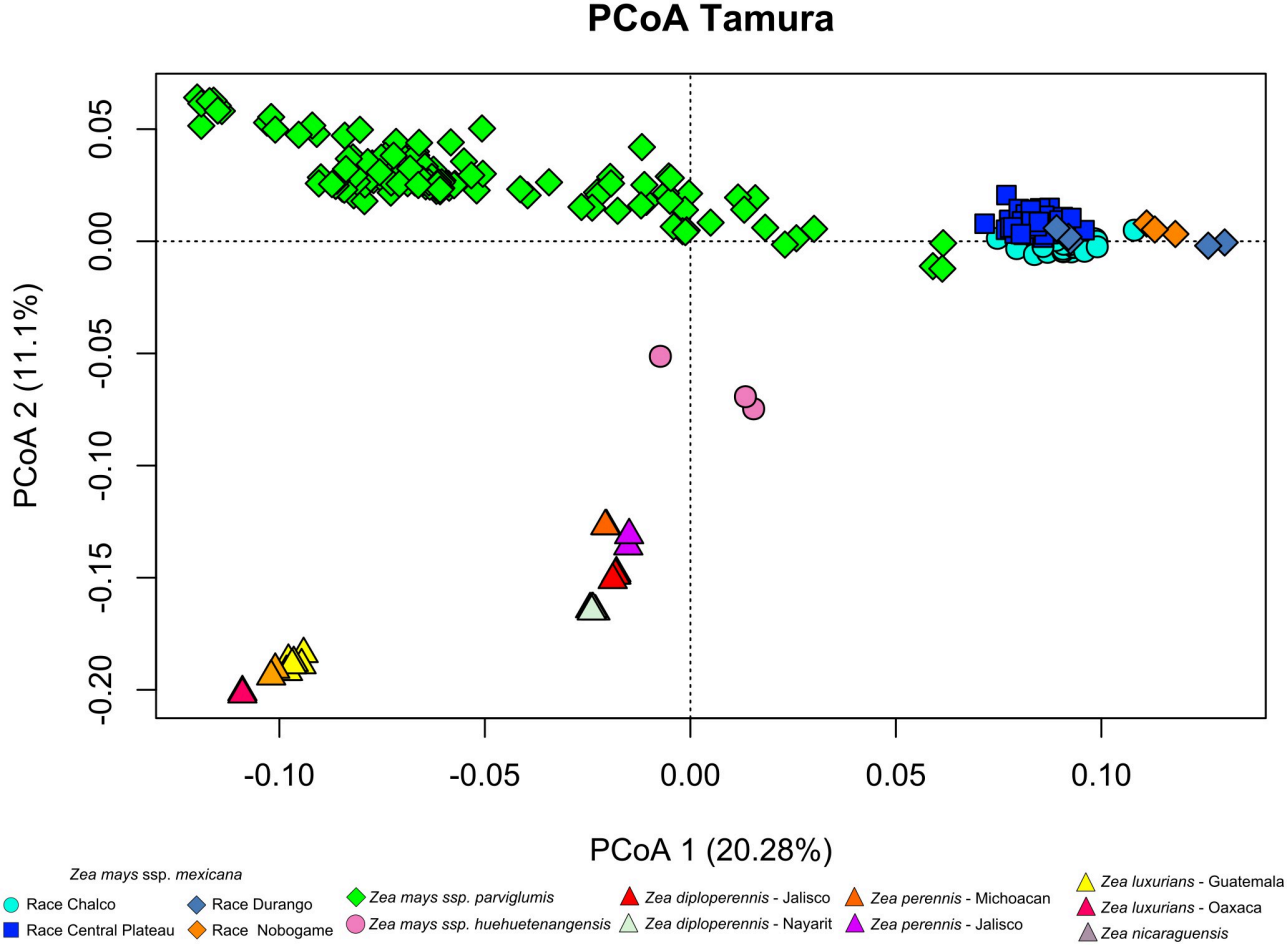
Plot of the two principal coordinate analyses showing the spatial differentiation among 276 populations of teosinte based on 33,929 SNPs.

The first three coordinates of the PCoA explain 36.22% of the variation with clustering of individuals corresponding to taxonomy and geographic distribution (Fig 6). The first coordinate (explaining 20.28% of the variation) separates the populations of the ssp. *parviglumis* and spp. *mexicana* following a geographic gradient. Coordinate 2 (explaining 11.1% of the variation) divides the genus into the *Luxuriantes* and *Zea* sections, with ssp. *huehuetenangensis* as intermediate. Plotting coordinates 2 and 3 shows similar patterns, but with higher separation of the races that make up ssp. *mexicana* (S1 and S2 Figs).

A first analysis of molecular variance (AMOVA) included the 276 populations divided into thirteen taxonomic groups (species, subspecies and races). The results show that there is a great genetic differentiation in all the levels considered which were significant (P <0.001). Of the total genetic variation, the highest percentage (62.51%) corresponds to the variation within individuals and the lowest variation was found between individuals within the same sampling locations (9.87%) (Table 1).

**Table 1:** AMOVA results of taxonomic groups and population of teosinte.

Source of variation	d.f.	Variance components	% of variation	*F* Statistics	*P value*
**Among Taxa**	12	613.18	14.46	*F*_IS_ 0.144	0.00
**Among populations within taxa**	263	558.3	13.16	*F*_SC_ 0.153	0.00
**Among individuals within populations**	3328	418.61	9.87	*F*_IS_ 0.136	0.00
**Among individuals**	3604	2651.59	62.51	*F*_IT_ 0.374	0.00

**Fig 7:**
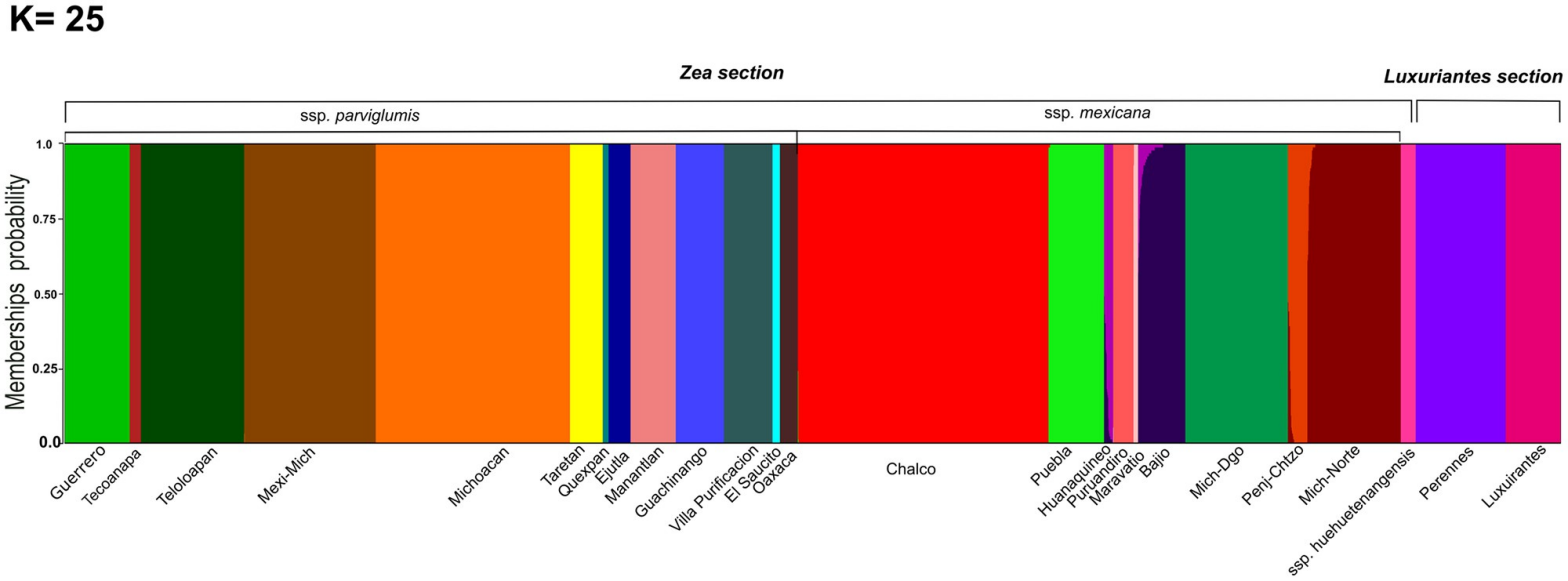
Discriminant analysis of principal components (DAPC). Barplot showing the probabilities of assignment of individuals to K = 25 genetic DAPC clusters.

Since the first AMOVA was based in current taxonomy, a second model to investigate the population hierarchy, was based on discriminant analysis of principal components (DAPC). In the DAPC analysis, in K = 25 was found to show the lowest levels of BIC (S3 Fig). The first 13 clusters correspond to ssp. *parviglumis* populations, followed by 9 clusters of ssp. *mexicana*, and an independent group for ssp. *huehuetenangensis*. The last two clusters group *Z*. *perennis* and *Z*. *diploperenis* in one, and *Z*. *luxurians* and *Z*. *nicaraguensis* in another (Fig 7).

The second AMOVA based on DAPC was similar to the first model; the percentage of variation among the 25 groups was 15.97%; higher than for the first model, while variation among groups was higher in the first model (Table 2).

**Table 2:** AMOVA results of 25 groups of DAPC and population of teosinte.

Source of variation	d.f.	Variance components	Percentage of variation	*F* Statistics	*P* value
**Among DAPC groups**	24	6254.86	15.97	*F*_IS_ 0.136	0.00
**Among populations within DAPC groups**	251	375.55	9.16	*F*_SC_ 0.108	0.00
**Among individuals within populations**	3,328	418.61	10.21	*F*_IS_ 0.159	0.00
**Among individuals**	3,604	2651.59	64.66	*F*_IT_ 0.353	0.00

**Fig 8:**
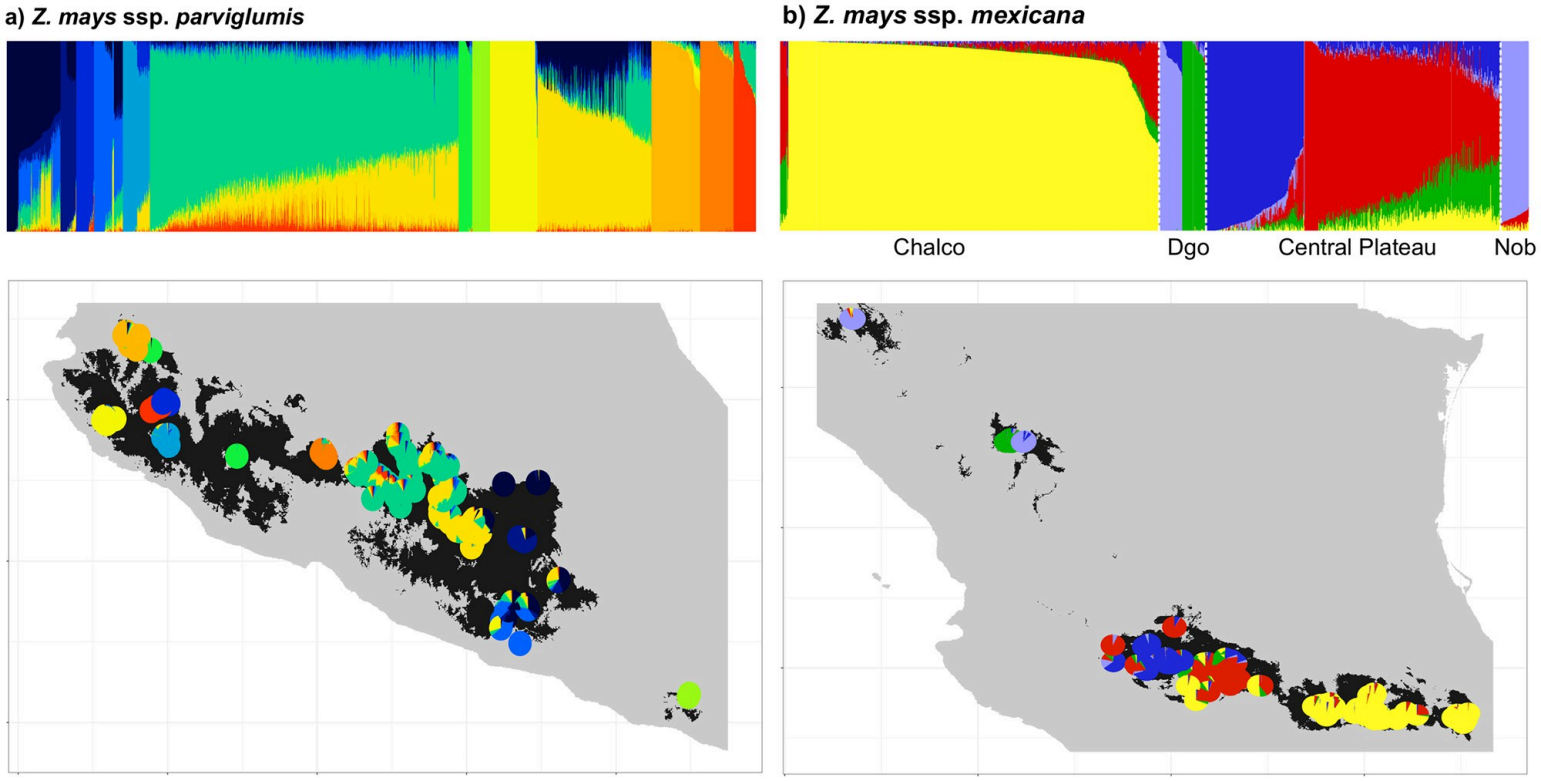
Admixture analyses for a) *Z*. *mays* ssp. *parviglumis* y b) *Z*. *mays* ssp. *mexicana* assuming K = 13 and K = 5 genetic clusters, respectively. In top panels each bar represents the proportion of different genetic clusters (colors) conforming an individual. For ssp. *parviglumis* individuals are ordered by genetic cluster, while for ssp. *mexicana* individuals are grouped according to the races Chalco, Central Plateau, Durango (Dgo) and Nobogame (Nob) separated by white dashed lines. Bottom panels show the potential distribution model of each taxa [20] overlaying the proportion of each genetic cluster by sampling locality plotted using pie charts.

According to the Admixture analysis the distribution of genetic variation within ssp. *parviglumis* follows a longitudinal gradient but with some clearly differentiated populations particularly at the West and East of its range (assuming K = 13, Fig 8a). In ssp. *mexicana* race Chalco is mostly differentiated in a genetic cluster, while race Central Plateau shows a West-East structure. Race Durango and Nobogame do not match independent genetic clusters (assuming K = 5, Fig 8b).

## Discussion

Most previous studies on *Zea* genetic variation used few individuals per taxon and did not sampled populations across the entire range of species, despite that the genus *Zea* is composed of only a handful of species, unlike other genera of Poaceae much richer [50]. Except for *Zea vespertilio*, here all teosinte taxa were sampled with a large number of individuals and populations to properly represent the genetic variation and structure within the genus. This was unfeasible a few years ago, however reduced representation genomic methods now make this possible [38, 51], opening the possibility to monitor populations at the genetic level, which is a long standing pending for conservation biology [52]. Additionally, our genetic results can be coupled with other studies including morphological [18] and environmental [16] evidence of population differentiation within teosintes, to better discuss conservation, management and use implications. Thus, we aim for the genetic results and data shown here to provide a base-line for long-term genetic monitoring and conservation actions to be implemented in teosintes.

### Low levels of genetic diversity within populations of conservation concern

Must studies on teosintes genetic diversity have focused on ssp. *parviglumis* and ssp. *mexicana* [31, 32, 34, 52, 53]. Studies including other taxa [26, 54, 55] averaged genetic diversity values by species or races, without providing the data of the individual populations, thus making it difficult to use this information for planning *in situ* or *ex situ* conservation actions at the population level. Former studies have also used a range of markers, including isoenzimes [56], Sanger sequencing of few nuclear and chloroplast loci [53, 57], microsatellites [26] and large SNPs datasets [31, 32, 34]. Therefore, it is difficult to compare previously estimated levels of genetic diversity among them or with the present results. However, in general terms, the former studies have shown that the highest levels of genetic diversity are found in ssp. *parviglumis* and ssp. *mexicana*. We found similar results when averaging at the taxon level, with ssp. *parviglumis* showing the highest diversity values followed by the Chalco and Central Plateau races of ssp. *mexicana*. The values for the races Durango, Nobogame of ssp. *mexicana* and *Z*. *perennis* present intermediate values. Lastly, ssp. *huehuetenangensis* and the species of the *Luxuriantes* section show the lowest values of diversity (Figs 3 and 4). This pattern is expected because ssp. *parviglumis* and ssp. *mexicana* are widely distributed taxa, while the rest of the teosintes occur only in few isolated and relatively small populations (Fig 2). However, our population level assessment shows that there are important differences in the level of genetic diversity within populations of all seven taxa, with many populations showing lower levels of genetic variation than expected, even within ssp. *parviglumis* and ssp. *mexicana* (Fig 4). Most of ssp. *parviglumis* nearly-continuous populations have become fragmented because of cattle farming and the establishment of pastures; however, the known largest wild populations in the Teloloapan-Zacatlancillo area in Guerrero, Huetamo-Morelia in Michoacán and Tejupilco-Palmar Chico in the State of México have the highest values of genetic diversity statistics registered in this study. On the other hand, populations of Jalisco and Oaxaca have the lowest levels of genetic diversity.

Within ssp. *mexicana* the populations that showed the highest levels of genetic variation are located Central Mexico (Fig 4). There, teosinte populations grow most commonly within maize fields (mostly of smallholders rainfed agriculture) and are common in disturbed surrounding areas, growing as weeds (Fig 1c). Most weedy teosintes resemble local maize races and natural hybrids can be found. However, within these same taxa there are cases of low genetic diversity, in particular populations of the races Nobogame and Durango. In both cases these populations correspond to the geographic border of the taxa, and the Oaxacan (ssp. *parviglumis*) and Nobogame and Durango (ssp. *mexicana*) populations are considerably isolated from the rest of the distribution, likely by natural historical processes. A previous study [54] also found reduced genetic diversity at the limits of the geographic distribution of ssp. *parviglumis* and ssp. *mexicana* and determined that it was caused by genetic drift. Interestingly, other populations showing relatively low genetic diversity in that study are at the limits of the environmental niche of the taxon, and thus where natural selection is driving local adaptation [58]. Therefore, although we found populations with reduced diversity compared to other teosinte populations, their adaptive value should not be underestimated. Also, it should be considered that the GBS dataset is biased towards the variation found in the references used for SNP calling, and thus there may be variation private to some populations that is not represented in the present analyses.

### Genetic diversity is geographically structured among and within teosinte taxa

The distribution of genetic variation within the *Zea* genus is highly structured, with genetic clusters generally matching taxonomic groups, but also subdivided in further clusters driven by geography (Figs 6 and 7). For the conventional taxonomy-based division of 13 taxa, the AMOVA showed significant variation between the different levels of structure of the 276 populations, with the greatest variation occurring within populations of the same taxon (Table 2). When the 276 populations were grouped into 25 genetic groups from the DAPC, the greatest variation was found within genetic groups (Table 2). Our extensive population sampling allows us to examine population structure within almost all teosinte taxa, and with the largest coverage of their distribution ranges up to today.

Within the *Luxuriantes* section, genetic diversity is structured in two clusters (Fig 7). A single group is formed by the perennial species (*Z*. *perennis* and *Z*. *diploperennis*), which are distributed in western Mexico mountain ranges. The second group is formed by *Z*. *luxurians* from Guatemala and Oaxaca, and by *Z*. *nicaraguensis*. Within the genetic clusters of *Z*. *luxurians* and *Z*. *diploperennis*, there is further genetic differentiation driven by geographic distance (Fig 5). As for the *Zea* section, ssp. *huehuetenangensis* forms an independent cluster from ssp. *parviglumis* and ssp. *mexicana*, and also from the teosintes of the *Luxuriantes* section, which are distributed in the same region of Central America. Loáisiga et al. [58] found a similar pattern, so our higher resolution data confirms that ssp. *huehuetenangensis* has likely remained naturally isolated for long periods of time.

The other two subspecies of the *Zea* section have the widest distribution of all teosintes and show strong geographic structure. On one hand ssp. *parviglumis* is subdivided in 13 genetic groups (Figs 7 and 8). The following restricted and isolated populations (which are the same showing the smallest levels of diversity, Fig 5) form their own genetic cluster, with little or no admixture with other clusters: Tecoanapa and Teloloapan from Guerrero, Taretan from Michoacán and all the populations of Jalisco (Guachinango, Ejutla, El Saucito, Manantlán and Villa Purificación) as well as the population from Oaxaca. On the opposite, other populations from Guerrero as well as those of the south of the Valley of Mexico City and Michoacán, are highly admixed following a longitudinal gradient (Fig 8). This gradient of differentiation within the central part of the range of the taxon is reflected in the IBD analysis (Fig 5). A similar pattern of IBD combined with highly differentiated populations was also found by Aguirre-Liguori et al. [54]. Genetic structure between populations of Jalisco and populations from other relatively close areas from western and central Mexico has been also recurrently found in other taxa of unrelated taxonomic groups inhabiting at different altitudes, for instance cloud forest shrubs [59], highland rattlesnakes [60], pines [61] and pine fungal endophytes [62]. Geologically, this is a topographically complex area known as “Jalisco Block”, which formed independently from the Sierra Madre Occidental and the Transmexican Volcanic Belt, mostly during the Miocene [63]. Today, two biogeographic regions confluence in this region [64], making biodiversity patterns particularly interesting. At the same time, this is one of the parts of Mexico where natural ecosystems have been more significantly modified by extensive agriculture (maize and avocado, mostly) and cattle raising [65].

On the other hand, ssp. *mexicana* is divided into several genetic groups that do not entirely match the taxonomic races it has been subdivided. In north west Mexico, the Durango race was subdivided into distinctive groups by both the PCoA, DAPC and Admixture analyses, one clustering closer to the Nobogame race in Chihuahua and the other to the Central Plateau race in central Mexico (Figs 6–8). This is particularly noteworthy given that the Durango populations are very close to each other (around 36 km), and considerably far from Nobogame and Central Plateau populations. Interestingly, several phylogeographic studies have found a similar break in the distribution of genetic diversity in this part of the Sierra Madre Occidental. However, there is no clear geographic barrier or historical geologic or climatic processes that could be associated with this pattern. What remains clear is that ssp. *mexicana* populations from north and central Mexico have remained naturally isolated for long periods of time, likely even during the Pleistocene climate fluctuations [66]. As for the races in the central part of Mexico, the Chalco race forms a relatively uniform genetic cluster, except from populations from Puebla which are admixed with one of the genetic clusters of Central Plateau (Figs 7 and 8). Central Plateau race shows more genetic structure, because there is a West-East pattern of differentiation (Fig 8) where populations could be divided in three clusters within the state of Michoacán (Cuitzeo, Indaparapeo and Churintzio; Fig 7).

### Conservation implications

According to the most recent risk assessment of Mesoamerican crop wild relatives [6] teosinte taxa comprised by a few isolated populations, like *Z*. *perennis* and ssp. *mexicana* race Nobogame, are at the highest risk (i.e. “critically endangered”), while widely distributed taxa, like ssp. *parviglumis*, are at the smallest risk (“least concern”, S1 File). Risk assessments, and thus conservation actions, are normally done at the species level, so it is an improvement that teosintes taxa were evaluated including infraspecific levels like subspecies and races. However, conserving genetic diversity requires actions at even higher resolution within taxa, that is, at the level of groups of distinct populations, which should be defined including genetic, morphological and ecological differences, as well as rates of disappearance, current threats and how (if) people manage local populations [67]. The former subdivision within taxa allows the representation of populations that are not interchangeable from a genetic or ecological point of view on a recent time scale. Additionally, for urgent conservation actions, populations with low values of genetic diversity and high values of genetic differentiation should be prioritized [68]. Coupling our genetic analyses with former morphological and ecogeographic studies [16, 18] shows that undertaking conservation actions at the taxon level alone would not be enough to truly represent teosinte genetic diversity. For instance, although ssp. *parviglumis* is listed as “least concern” in the IUCN [S1 File] risk assessment, genetically it is subdivided in 13 clusters (Figs 7 and 8), with some populations forming highly differentiated clusters and low levels of genetic variation (Fig 5). Thus, populations should be monitored, conserved and in some cases restored based on idiosyncratic aspects, including their genetic, ecological and morphological differentiation, possible role on maize domestication and current threats by human activities.

Due to very low values of diversity and marked differentiation with respect to other populations and taxa, populations of highest conservation concern belong to *Z*. *perennis* and *Z*. *diploperennis* of the *Luxuriantes* section, and are distributed within Mexico. Within *Z*. *perennis*, Michoacan and Jalisco populations should be considered independently. Among these, the Jalisco populations are highly threatened because even if they are located within the Nevado de Colima Volcano National Park, the areas where they grow were recently invaded by avocado plantations. Within *Z*. *diploperennis*, populations from Jalisco and Nayarit show low and very low levels of genetic diversity, respectively (Figs 3 and 4), as well as isolation by distance (Fig 5), so these populations should also be considered independently for conservation purposes. Before being evaluated by the IUCN risk assessment, *Z*. *perennis* and *Z*. *diploperennis* were already listed in 2010 in the NOM-059 (Mexican red list of threatened species [S1 File]) as in critical danger and threatened, respectively. The results of the present work show that conservation measurements for the protection of these taxa have not been sufficient, so it is urgent to implement different actions. Conservation of *Z*. *luxurians*, also of the *Luxuriantes* section, should include ad independent groups the populations of Oaxaca (Mexico), Nicaragua and Guatemala, due to their very low and low levels of genetic diversity, respectively (Figs 3 and 4) and isolation by distance (Fig 5).

Within the *Zea* section there are also populations of high concern, both considering their genetic differentiation and human induced threats. These are ssp. *huehuetenangensis*, and the most differentiated and geographically restricted genetic clusters of ssp. *mexicana* and ssp. *parviglumis*. These subspecies were not considered threatened by the NOM-059 in 2010, because the evaluation was made at the species level, at which population sizes are considerably high. However, the more recent assessment by the IUCN in 2017 evaluated subspecies and races independently (S1 File). Subspecies *huehuetenangensis* is endangered, showing the smallest levels of genetic diversity of all *Zea mays* teosintes (Figs 3 and 4) and its own highly differentiated genetic cluster (Figs 6 and 7). Within ssp. *mexicana*, the populations of highest conservation concern belong to the races Nobogame and Durango (Nombre de Dios populations), which showed the lowest levels of genetic diversity within ssp. *mexicana* (Fig 3); they form independent genetic clusters (Figs 6–8) and have a restricted distribution. Race Durango is listed as endangered by the IUCN Red List. But for conservation purposes our genetic data shows that this race should be divided in two genetic clusters (Fig 8), which means that population sizes of these groups should be smaller than considering the race as a unit, thus increasing their risk. Race Nobogame was listed as critically endangered, and our genetic data agrees that for conservation purposes populations of this race should be considered as a unit. Subspecies *parviglumis* was listed as least concern by the IUCN, however there are populations of high conservation concern within it, due to their isolation, genetic differentiation and relatively low genetic diversity (Figs 4, 7 and 8). These are distributed in Guerrero (Tecoanapa and Teloloapan), Michoacán (Taretan), Jalisco (Guachinango, Ejutla, El Saucito, Manantlán and Villa Purificación) and Oaxaca (San Cristobal Honduras).

Populations of the largest genetic clusters include races Chalco and Central Plateau from ssp. *mexicana* and the eastern and Balsas Basin distribution of ssp. *parviglumis*. Populations of these clusters should also be considered as independent units for conservation purposes. Populations of these genetic clusters are of relatively less concern because their distributions and levels of genetic diversity are the largest within the genus (Fig 4). However, representing their genetic diversity in conservation actions is challenging because it is distributed in longitudinal gradients (Fig 8), which are difficult to fully cover by few sampling localities. Additionally, these populations grow very close, or even within, maize fields, including local landraces and modern maize varieties (Fig 1c–1e). This sympatric distribution has shown to produce gene flow from maize modern varieties into teosintes, particularly into ssp. *mexicana* and to a lesser extent (in samples from the 1980s decade) into ssp. *parviglumis* [30].

As for taking conservation of teosintes genetic diversity into practice, both *in situ* and *ex situ* actions are required. However, considering that most populations of teosinte taxa are distributed within regions of high social conflict and presence of organized crime [69], *ex situ* conservation actions are particularly urgent, while social conditions for *in situ* conservation became better in key areas where the smallest populations are distributed. Based on previous teosinte collection work in Mexico [24, 70] in most cases it is possible to sample all populations with sample sizes of at least 500 individuals and around from 5,000 to 10,000 seeds. The idea is to spend the minimum amount of time and effort in each locality trying to obtain all the common alleles, visiting as many different sites as possible to maximize the possibility of capturing new adaptive variants. For seed collection of population clusters where populations have high levels of genetic diversity and low levels of differentiation, the suggested general strategy is to collect from a few sites, but a large number of individuals per population. For genetic clusters where populations show low levels of diversity and high values of differentiation, it would be recommended collecting a few plants per population and a large number of populations. In the case of small populations with less than 200 individuals, sampling must be done very carefully, obtaining one or two seeds per plant, and ensuring their *ex situ* regeneration in greenhouses for later recovery *in situ*.

*In situ* conservation actions are more challenging, because there are several uncontrollable environmental factors (droughts, floods, fires, etc.) as well as human factors such as farmer’s management decisions and land use changes. *In situ* conservation actions should be thus further discussed and proposed in the social and environmental conditions of each region. What remains clear from the current study, is that some of the reduced levels of genetic diversity found are caused by natural processes driven by historical isolation and adaptation, but that also recent anthropic activities play a role. Some of the naturally isolated and relatively small populations within all teosinte taxa have been further fragmented by the growth of intensive agriculture (mostly of maize and avocado), opening of non-native grasslands for livestock and urbanization [12, 16, 25] (Fig 1d and 1f). In these situations, education and outreach activities are needed, so that farmers know of the present and future value of conserving teosintes and can modify their agronomic practices to protect them. Although there is no quantitative data available, qualitative monitoring during more than 50 years [16, 71] allows us to detect that teosintes habitat is decreasing, and thus their population sizes, which in turn can considerably reduce variation by genetic drift. Monitoring and estimating local population sizes and estimating effective population sizes, as has recently been proposed to do far as many taxa as possible [72] thus becomes a key next step for teosinte *in situ* conservation. The effectiveness of ongoing protection measurements on some taxa should also be evaluated. Importantly, even if the currently work represents the most updated and representative teosinte sampling, most of it is more than 10 years old (S1 Data). Thus, part of the diversity described here may have been already been lost, especially for populations that were already too small a decade ago.

Finally, in order to successfully achieve an action plan for *in situ* conservation, it is necessary for the academic sectors and governments at all levels, as well as local communities, to undertake environmental education programs and the dissemination of information on the importance of teosintes, so that local agricultural activities can co-exist with teosintes. This could be done through the new public policies on agroecology and agrobiodiversity conservation that the Mexican government is developing [73], if teosinte conservation is incorporated into them. The timing for teosinte conservation matters. Teosinte’s genetic diversity can contribute to overcome the challenges that future generations of maize farmers will face, but without prompt conservation actions it is likely that these genetic diversity would be lost.

## Conclusions

Empirical genetic data is needed to propose detailed strategies for genetic diversity conservation. For this, GBS is useful because it allows genotyping tens of individuals per population, totalling thousands of individuals spanning the entire range of several species. This sampling allowed to determine the levels of differentiation among populations and taxa, as well as the levels of genetic diversity. However, genetic data alone is not enough to define conservation strategies: it is necessary to integrate local socioenvironmental conditions, as well as to incorporate taxonomic, morphological, physiological and ecological characteristics of each taxa, as done here.

Sampling through time allows to monitor indicators and determine whether or not conservation strategies are working. In this sense, this investigation includes samples collected at least a decade ago, which opens the possibility of repeating the sampling to compare different time points, and thus it is the foundation of a monitoring program. Likewise, this study includes all *Zea* species present in Mexico, including poorly studied taxa. We hope the present study to serve as example to the type of research needed in other Mesoamerican crop wild relatives.

## Supporting information

S1 Fig Plot of the PCo1 and PCo3 of principal coordinate analyses. (PNG)

S2 Fig Plot of the PCo2 and PCo3 of principal coordinate analyses. (PNG)

S3 Fig Plot of value of BIC. (PNG)

S1 File Threat category of teosinte taxa according to the IUCN and Mexican red lists. (DOCX)

S1 Data Passport data of teosinte population. (XLSX)

S2 Data Pairwise matrix of F_*ST*_ values. (XLSX)

S3 Data Pairwise matrix of geographic distance values. (XLSX)

S4 Data Diversity genetic index values by teosinte population and summary by taxa. (XLSX)

## References

[1] Dobson A, Lodge D, Alder J, Cumming GS, Keymer J, Mcglade J, et al. Habitat loss, trophic collapse, and the decline of ecosystem services. Ecology. 2006. doi: 10.1890/0012-9658(2006)87[1915:hltcat]2.0.co;2 16937628

[2] Wilcove DS, Rothstein D, Dubow J, Phillips A, Losos E. Quantifying Threats to Imperiled Species in the United States. BioScience. 1998; 48: 607–615. doi: 10.2307/1313420

[3] Young A, Boyle T, Brown T. The population genetic consequences of habitat fragmentation for plants. Trends Ecol Evol. 1996; 11:413–8. doi: 10.1016/0169-5347(96)10045-8 21237900

[4] Frankham R. Genetics and conservation biology. C R Biol. 2003; 326:22–9. doi: 10.1016/s1631-0691(03)00023-4 14558445

[5] Oostermeijer J, Luijten S, Nijs JD. Integrating demographic and genetic approaches in plant conservation. Biol Conserv. 2003; 113:389–398. doi: 10.1016/s0006-3207(03)00127-7

[6] Goettsch B, Urquiza‐Haas T, Koleff P, Acevedo Gasman F, Aguilar‐Meléndez A, Alavez V, et al. Extinction risk of Mesoamerican crop wild relatives. Plants People Planet. 2021; 3(6): 775–795. doi: 10.1002/ppp3.10225

[7] Tobón-Niedfeldt W, Mastretta-Yanes A, Urquiza-Haas T, Goettsch B, Cuervo-Robayo AP, Urquiza-Haas E, et al. Incorporating evolutionary and threat processes into crop wild relatives conservation. 2022; Nature Communications, 13(1), 1–18. doi: 10.1038/s41467-022-33703-0 36271075 PMC9587227

[8] Schemske DW, Husband BC, Ruckelshaus MH, Goodwillie C, Parker IM, Bishop JG. Evaluating approaches to the conservation of rare and endangered plants. Ecology. 1994; 75(3): 584–606. doi: 10.2307/1941718

[9] Diario Oficial de la Federación. Acuerdo por el que se determinan Centros de Origen y Centros de Diversidad Genética del Maíz. DOF: 02/11/2012. https://dof.gob.mx/nota_detalle.php?codigo=5276453&fecha=02/11/2012#gsc.tab=0

[10] Iltis HH, Doebley JF. Taxonomy of *Zea* (Gramineae) II. Subspecific categories in the Zea mays complex and a generic synopsis. Amer J Bot. 1980; 67: 994–1004. doi: 10.1002/j.1537-2197.1980.tb07731.x

[11] Doebley JF, Iltis HH. Taxonomy of *Zea* (Gramineae) I. A subgeneric classification with key to taxa. Amer J Bot. 1980; 67: 982–993. doi: 10.1002/j.1537-2197.1980.tb07730.x

[12] Doebley JF. Molecular evidence and the evolution of maize. Econ Bot. 1990; 44: 6–27. doi: 10.1007/BF02860472

[13] Wilkes HG. Teosinte: the closest relative of maize revisited. Maydica. 1985; 30(2): 209–223.

[14] ltis HH, Benz BF. *Zea nicaraguensis* (Poaceae), a new teosinte from Pacific coastal Nicaragua. Novon. 2000; 10: 382–390. doi: 10.2307/3392992

[15] Gómez-Laurito J. A new species of Zea (Poaceae) from the Murciélago Islands, Santa Elena Peninsula, Guanacaste, Costa Rica. Brenesia. 2013; 80: 36–39

[16] Sánchez González JD, Ruiz Corral JA, García GM, Ojeda GR, Larios LD, Holland JB, et al. Ecogeography of teosinte. PLoS ONE. 2018; 13 (2): e0192676. doi: 10.1371/journal.pone.0192676 29451888 PMC5815594

[17] Sánchez GJJ, Kato YTA, Aguilar SM, Hernández CJM, López RA, Ruiz CJA. Distribución y caracterización del teocintle. Libro Técnico Núm. 2. Centro de Investigación Regional del Pacífico Centro, Instituto Nacional de Investigaciones Forestales, Agrícolas y Pecuarias. 1998; 150p.

[18] Rivera-Rodríguez DM, Sánchez González JJ, De la Cruz Larios L, Santacruz-Ruvalcaba F, Ruiz Corral JA. Morphological and climatic variability of teosinte (*Zea* spp.) and relationships among taxa. Syst Bot. 2019; 44(1):41–51. doi: 10.1600/036364419X697886

[19] Mano Y, Omori F. Breeding for flooding tolerant maize using "teosinte" as a germplasm resource. Plant root. 2007; 1:17–21. doi: 10.3117/plantroot.1.17

[20] Mano Y, Omori F. Flooding tolerance in interspecific introgression lines containing chromosome segments from teosinte (*Zea nicaraguensis*) in maize (*Zea mays* subsp. *mays*). Ann Bot. 2013; 112(6):1125–39. doi: 10.1093/aob/mct160 23877074 PMC3783227

[21] Nault LR. Origins of leafhopper vectors of maize pathogens in Mesoamerica. In DT Gordon, JK Knoke, LR Nault and RM Ritter [Eds]. Proceedings International Maize Virus Disease Colloquium and Workshop, 2–6 August 1982, pp. 75–82. The Ohio State University, Ohio Agricultural Research and Development Center, Wooster. 1983. USA.

[22] Amusan IO, Rich PJ, Menkir A, Housley T, Ejeta G. Resistance to Striga hermonthica in a maize inbred line derived from *Zea diploperennis*. New Phytol. 2008; 178(1):157–66. doi: 10.1111/j.1469-8137.2007.02355.x 18208472

[23] Beadle GW. The ancestry of corn. Scientific American. 1980; 242(1): 112–119. doi: 10.1038/scientificamerican0180-112

[24] Sánchez G. JJ, Ordaz LS. El Teocintle en México. Distribución y situación actual de las poblaciones. Rome: IBPGR Press; 1987.

[25] Sánchez GJJ, Ruiz CJA. Teosinte Distribution in Mexico. In: Serratos JA., Willcox MC. Castillo y F. (eds.). Gene Flow Among Maize Landraces, Improved Maize Varieties, and Teosinte: Implications for Transgenic Maize. Mexico, D.F. CIMMYT. 1997; pp.18–35.

[26] Fukunaga K, Hill J, Vigouroux Y, Matsuoka Y, Sanchez G J, Liu K, et al. Genetic diversity and population structure of teosinte. Genetics. 2005; 169(4):2241–54. doi: 10.1534/genetics.104.031393 15687282 PMC1449573

[27] Wang P, Lu Y, Zheng M, Rong T, Tang Q. RAPD and internal transcribed spacer sequence analyses reveal Zea nicaraguensis as a section *Luxuriantes* species close to *Zea luxurians*. PLoS One. 2011; 6: e16728. doi: 10.1371/journal.pone.0016728 21525982 PMC3078115

[28] Sánchez G JJ, De La Cruz LL, Vidal M VA, Ron PJ, Taba S, Santacruz‐Ruvalcaba F, et al. Three new teosintes (*Zea* spp., Poaceae) from México. Am J Bot. 2011; 98(9):1537–48. doi: 10.3732/ajb.1100193 21875968

[29] Buckler ES, Holtsford TP. *Zea* systematics: ribosomal ITS evidence. Mol Biol Evol. 1996; 13(4):612–22. doi: 10.1093/oxfordjournals.molbev.a025621 8882504

[30] Rojas-Barrera IC, Wegier A, Sanchéz González JJ, Owens GL, Rieseberg LH, Piñero D. Contemporary evolution of maize landraces and their wild relatives influenced by gene flow with modern maize varieties. Proc Natl Acad Sci. 2019; 116(42): 21302–21311. doi: 10.1073/pnas.1817664116 31570572 PMC6800366

[31] van Heerwaarden J, Doebley J, Briggs WH, Glaubitz JC, Goodman MM, Gonzalez JD, et al. Genetic signals of origin, spread, and introgression in a large sample of maize landraces. Proc Natl Acad Sci. 2011; 108(3):1088–92. doi: 10.1073/pnas.1013011108 21189301 PMC3024656

[32] Hufford MB, Lubinksy P, Pyhäjärvi T, Devengenzo MT, Ellstrand NC, Ross-Ibarra J. The genomic signature of crop-wild introgression in maize. PLoS Genet. 2013; 9(5):e1003477. doi: 10.1371/journal.pgen.1003477 23671421 PMC3649989

[33] Pyhäjärvi T, Hufford MB, Mezmouk S, Ross-Ibarra J. Complex patterns of local adaptation in teosinte. Genome Biol Evol. 2013; 5(9):1594–609. doi: 10.1093/gbe/evt109 23902747 PMC3787665

[34] Aguirre-Liguori JA, Gaut BS, Jaramillo-Correa JP, Tenaillon MI, Montes-Hernández S, Garcıa-Oliva F, et al. Divergence with gene flow is driven by local adaptation to temperature and soil phosphorus concentration in teosinte subspecies (*Zea mays parviglumis* and *Zea mays mexicana*). Mol Ecol. 2019; 2814–30. doi: 10.1111/mec.15098 30980686

[35] CONABIO. Proyecto Global de Maíces Nativos. Comisión Nacional para el Conocimiento y Uso de la Biodiversidad. 2011[cited 3 April 2023]. In: Biodiversidad Mexicana. Ciudad de México. https://biodiversidad.gob.mx/diversidad/proyectoMaices

[36] CIMMYT. 2006. Protocolos de laboratorio: Laboratorio de Genética Molecular Aplicada del CIMMYT. Tercera edición. México, D.F.: CIMMYT.

[37] Fick SE, Hijmans RJ. WorldClim 2: new 1km spatial resolution climate surface for global land areas. Int J Climatol. 2017; 37(12): 4302–4315. doi: 10.1002/joc.5086

[38] Elshire RJ, Glaubitz JC, Sun Q, Poland JA, Kawamoto K, Buckler ES, et al. A robust, simple genotyping-by-sequencing (GBS) approach for high diversity species. PLoS One; 2011; 6(5):e19379. doi: 10.1371/journal.pone.0019379 21573248 PMC3087801

[39] Glaubitz JC, Casstevens TM, Lu F, Harriman J, Elshire RJ, Sun Q, et al. TASSEL-GBS: A High Capacity Genotyping by Sequencing Analysis Pipeline. PLoS One. 2014; 9(2): e90346. doi: 10.1371/journal.pone.0090346 24587335 PMC3938676

[40] Chang CC, Chow CC, Tellier LC, Vattikuti S, Purcell SM, Lee JJ. Second-generation PLINK: rising to the challenge of larger and richer datasets. Gigascience. 2015; 4(7). doi: 10.1186/s13742-015-0047-8 . 25722852 PMC4342193

[41] Benazzo A, Panziera A, Bertorelle G. 4P: fast computing of population genetics statistics from large DNA polymorphism panels. Ecol Evol. 2015; 5:172–175. doi: 10.1002/ece3.1261 25628874 PMC4298444

[42] Canteri E, Fordham DA, Li S, Hosner PA, Rahbek C, Nogués‐Bravo D. IUCN Red List protects avian genetic diversity. Ecography: pattern and diversity in ecology. 2021; 44(12):1808–181. doi: 10.1111/ecog.05895

[43] Rozas J, Ferrer-Mata A, Sánchez-Del Barrio JC, Guirao-Rico S, Librado P, Ramos-Onsins SE, et al. DnaSP v6: DNA Sequence Polymorphism Analysis of Large Datasets. Mol Biol Evol. 2017; 34: 3299–3302. doi: 10.1093/molbev/msx248 29029172

[44] R Core Team. R: A language and environment for statistical computing. 2021; R Foundation for Statistical Computing, Vienna, Austria. URL: https://www.R-project.org/.

[45] Excoffier L, Lischer H. Arlequin v.3.5, An Integrated Software Package for Population Genetics Data Analysis. 2015; Switzerland: University of Berne.

[46] Jombart T. *adegenet*: a R package for the multivariate analysis of genetic markers. Bioinformatics. 2008; 24: 1403–1405. doi: 10.1093/bioinformatics/btn129 18397895

[47] Kumar S, Stecher G, Tamura K. MEGA7: Molecular Evolutionary Genetics Analysis Version 7.0 for Bigger Datasets. Mol Biol Evol. 2016; 33:1870–1874. doi: 10.1093/molbev/msw054 27004904 PMC8210823

[48] Rohlf FJ. NTSYSpc: Numerical taxonomy system. ver. 2.21 c. 2009; Setauket, New York: Exeter Software.

[49] Alexander DH, Novembre J, Lange K. Fast model-based estimation of ancestry in unrelated individuals. Genome research. 2009; 19(9): 1655–1664. doi: 10.1101/gr.094052.109 19648217 PMC2752134

[50] Soreng RJ, Peterson PM, Romaschenko K, Davidse G, Zuloaga FO, Judziewicz EJ, et. al. A worldwide phylogenetic classification of the Poaceae (Gramineae). J Syst Evol. 2015; 53(2), 117–137. doi: 10.1111/jse.12150

[51] Poland JA, Rife TW. Genotyping‐by‐sequencing for plant breeding and genetics. The Plant Genome. 2012; 5(3): 92–102. doi: 10.3835/plantgenome2012.05.0005

[52] Hoban S, Campbell CD, da Silva JM, Ekblom R, Funk WC, Garner BA, et al. Genetic diversity is considered important but interpreted narrowly in country reports to the Convention on Biological Diversity: Current actions and indicators are insufficient. Biol Conserv. 2021; 261, 109233. doi: 10.1016/j.biocon.2021.109233

[53] Moeller DA, Tenaillon MI, Tiffin P. Population structure and its effects on patterns of nucleotide polymorphism in teosinte (*Zea mays* ssp. *parviglumis*). Genetics. 2007; 176: 1799–1809. doi: 10.1534/genetics.107.070631 17483429 PMC1931540

[54] Aguirre-Liguori JA, Tenaillon MI, Vázquez-Lobo A, Gaut BS, Jaramillo-Correa JP, Montes-Hernandez S, et al. Connecting genomic patterns of local adaptation and niche suitability in teosintes. Mol Ecol. 2017; 26: 4226–4240. doi: 10.1111/mec.14203 28612956

[55] Doebley JF, Goodman MM, Stuber CW. Isoenzymatic variation in *Zea* (Gramineae). Syst Bot. 2984; 9: 203–218. doi: 10.2307/2418824

[56] Loáisiga CH, Brantestam AK, Diaz O, Salomon B, Merker A. Genetic diversity in seven populations of Nicaraguan teosinte (*Zea nicaraguensis* Iltis et Benz) as estimated by microsatellite variation. Genet Resour Crop Evol. 2011; 58: 1021–1028. doi: 10.1007/s10722-010-9637-6

[57] Ross-Ibarra J, Tenaillon M, Gaut BS. Historical divergence and gene flow in the genus *Zea*. Genetics. 2009; 181(4), 1399–1413. doi: 10.1534/genetics.108.097238 19153259 PMC2666508

[58] Loáisiga CH, Rocha O, Brantestam AK, Salomon B, Merker A. Genetic diversity and gene flow in six accessions of Meso-America teosintes. Genet Resour Crop Evol. 2012; 59(1), 95–111. doi: 10.1007/s10722-011-9671-z

[59] Ornelas JF, González C. Interglacial genetic diversification of *Moussonia deppeana* (Gesneriaceae), a hummingbird‐pollinated, cloud forest shrub in northern Mesoamerica. Mol Ecol. 2014; 23(16), 4119–4136. doi: 10.1111/mec.12841 24954419

[60] Bryson RW Jr, Murphy RW, Graham MR, Lathrop A, Lazcano D. Ephemeral Pleistocene woodlands connect the dots for highland rattlesnakes of the Crotalus intermedius group. J Biogeogr. 2011; 38(12), 2299–2310. doi: 10.1111/j.1365-2699.2011.02565.x

[61] Moreno‐Letelier A, Piñero D. Phylogeographic structure of *Pinus strobiformis* Engelm. across the Chihuahuan Desert filter‐barrier. J Biogeogr. 2009; 36(1), 121–131. doi: 10.1111/j.1365-2699.2008.02001.x

[62] Salas-Lizana R, Santini NS, Miranda-Pérez A, Piñero DI. The Pleistocene glacial cycles shaped the historical demography and phylogeography of a pine fungal endophyte. Mycol prog. 2012; 11(2), 569–581. doi: 10.1007/s11557-011-0774-x

[63] Ferrari L, Orozco-Esquivel T, Manea V, Manea M. The dynamic history of the Trans-Mexican Volcanic Belt and the Mexico subduction zone. Tectonophysics. 2012; 522, 122–149. doi: 10.1016/j.tecto.2011.09.018

[64] Morrone JJ. Biogeographical regionalisation of the Neotropical region. Zootaxa. 2014; 3782(1), 1–110. doi: 10.11646/zootaxa.3782.1.1 24871951

[65] CONABIO. Capital natural y bienestar social. México: Comisión Nacional para el Conocimiento y Uso de la Biodiversidad Press; 2006.

[66] Hufford MB, Xu X, Van Heerwaarden J, Pyhäjärvi T, Chia JM, Cartwright RA. et al. Comparative population genomics of maize domestication and improvement. Nat Genet. 2012; 44(7), 808–811. doi: 10.1038/ng.2309 22660546 PMC5531767

[67] Frankham R, Ballou JD, Ralls K, Eldridge M, Dudash MR, Fenster CB, et al. Genetic management of fragmented animal and plant populations. New York: Oxford University Press: 2017. doi: 10.1093/oso/9780198783398.001.0001

[68] Hoban S, Schlarbaum S. Optimal sampling of seeds from plant populations for ex-situ conservation of genetic biodiversity, considering realistic population structure. Biol Conserv. 2014; 77, 90–9. doi: 10.1016/j.biocon.2014.06.014

[69] Fuertes Celis MP. Geografía de la violencia en México. Un acercamiento a la reconfiguración territorial de la violencia generada por el crimen organizado. 1st ed. Aguascalientes. Centro de Investigación y Docencia Económicas Press; 2016

[70] Sánchez G. JJ, Hernández CJM, Gómez MNO, Vidal MVA, De la Cruz LL, Miranda MM, et al. Distribución geográfica del Teocintle (Zea spp.) en México y situación actual de las poblaciones. Diversidad y distribución del maíz nativo y sus parientes silvestres en México, México: Biblioteca Básica de Agricultura Press; 2013.

[71] Wilkes GH. Urgent notice to all maize researchers: disappearance and extinction of the last wild teosinte population is more than half completed. A proposal for teosinte evolution and conservation in situ: the Balsas, Guerrero, Mexico. Maydica. 2007; 52: 49–70.

[72] Hoban S, Bruford MW, da Silva JM. et al. Genetic diversity goals and targets have improved, but remain insufficient for clear implementation of the post-2020 global biodiversity framework. Conserv Genet. 2023; 24: 181–191. doi: 10.1007/s10592-022-01492-0 36683963 PMC9841145

[73] Diario Oficial de la Federación. Programa Sectorial de Medio Ambiente y Recursos Naturales 2020-2024.DOF: 07/07/2020. https://www.dof.gob.mx/nota_detalle.php?codigo=5596232&fecha=07/07/2020#gsc.tab=0

