# Supplementary figures and images for: Genomic diversity and population structure of teosinte (Zea spp.) and its conservation implications
Source: PLoS One. 2023 Oct 11;18(10):e0291944. doi: 10.1371/journal.pone.0291944 (PMC10566683; doi:10.1371/journal.pone.0291944)

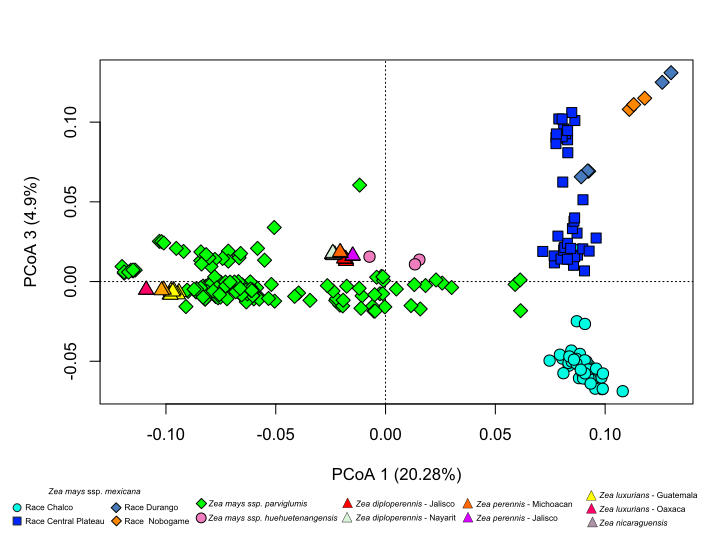

Supplement: S1 Fig — (PNG) [file pone.0291944.s001.png]

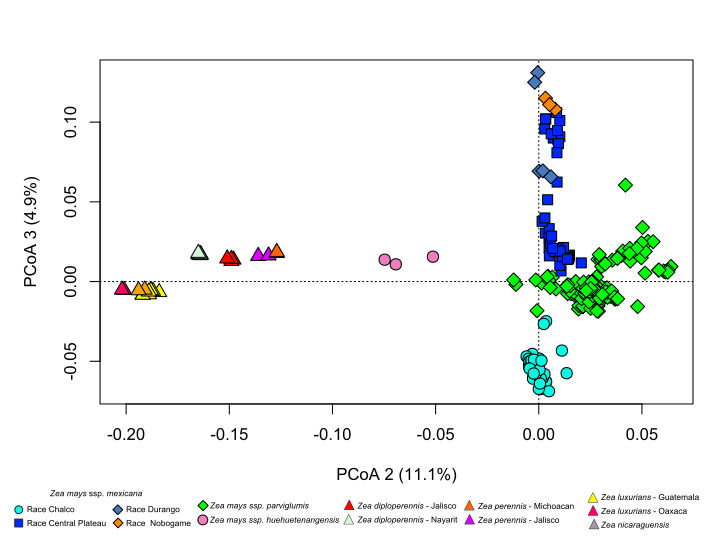

Supplement: S2 Fig — (PNG) [file pone.0291944.s002.png]

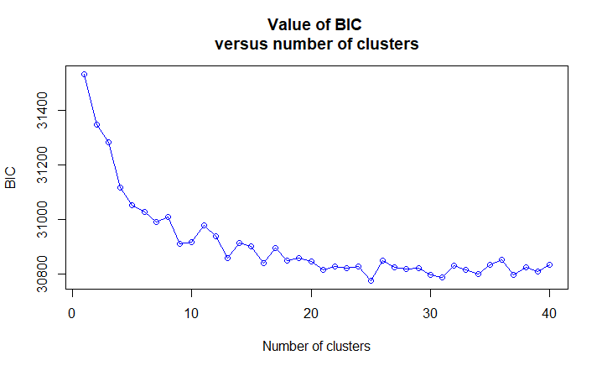

Supplement: S3 Fig — (PNG) [file pone.0291944.s003.png]
