# Supplementary material for: Genomic diversity and population structure of teosinte (Zea spp.) and its conservation implications
Source: PLoS One. 2023 Oct 11;18(10):e0291944. doi: 10.1371/journal.pone.0291944 (PMC10566683; doi:10.1371/journal.pone.0291944)
Supplement: S1 File — (DOCX) [file pone.0291944.s004.docx]

**S1 Table. Threat category of teosinte taxa according to the IUCN and Mexican red lists.**

| **Taxon** | **IUCN Red List Category (2017)** | **Mexican NOM-059 Red List (2010)** |
| --- | --- | --- |
| *Zea diploperennis* | Endangered [1] | Threatened [10] |
| *Zea luxurians* | Vulnerable [2] |  |
| *Zea mays* ssp. *huehuetenangensis* | Endangered [3] |  |
| *Zea mays* ssp. *mexicana* Chalco race | Least concern [4] |  |
| *Zea mays* ssp. *mexicana* Durango race | Endangered [5] |  |
| *Zea mays* ssp. *mexicana* Mesa-Central race | Least concern [6] |  |
| *Zea mays* ssp. *mexicana* Nobogame race | Critically endangered [7] |  |
| *Zea mays* ssp. *parviglumis* | Least concern [8] |  |
| *Zea perennis* | Critically endangered [9] | Extinction risk [10] |

Bibliography.

1. Aragón Cuevas F, Contreras A, de la Cruz Larios L, González Ledesma M, Ruíz Corral JA, Menjívar J, Sánchez JJ. *Zea diploperennis.* The IUCN Red List of Threatened Species 2019. 2019; e.T77726057A77726102. http://dx.doi.org/10.2305/IUCN.UK.2019- 2.RLTS.T77726057A77726102.en. Accessed on 20 July 2022.
2. Aragón Cuevas F, Menjívar J, Ruíz Corral JA, González Ledesma M, Contreras A, Azurdia C, de la Cruz Larios L, Sánchez JJ. *Zea luxurians.* The IUCN Red List of Threatened Species 2019. 2019; e.T77726182A77726358. https://dx.doi.org/10.2305/IUCN.UK.2019-2.RLTS.T77726182A77726358.en. Accessed on 20 July 2022.
3. Azurdia C, Sánchez JJ, Contreras A. *Zea mays* subsp. *huehuetenangensis* (amended version of 2019 assessment). The IUCN Red List of Threatened Species 2020. 2020; e.T109973611A175177730. https://dx.doi.org/10.2305/IUCN.UK.2020-3.RLTS.T109973611A175177730.en. Accessed on 20 July 2022.
4. de la Cruz Larios L, Ruíz Corral JA, Aragón Cuevas F, Contreras A, Sánchez J. *Zea mays* subsp. *mexicana* (Chalco subpopulation). The IUCN Red List of Threatened Species 2019. 2019; e.T111341717A111341727. https://dx.doi.org/10.2305/IUCN.UK.2019-2.RLTS.T111341717A111341727.en. Accessed on 20 July 2022.
5. Ruíz Corral JA, de la Cruz Larios L, Oliveros O, Contreras A, Sánchez JJ. *Zea mays* subsp. *mexicana* (Durango subpopulation). The IUCN Red List of Threatened Species 2019. 2019; e.T111341754A111341757. https://dx.doi.org/10.2305/IUCN.UK.2019-2.RLTS.T111341754A111341757.en. Accessed on 20 July 2022.
6. de la Cruz Larios L, Ruíz Corral JA, Oliveros O, Contreras A, Aragón Cuevas F, Sánchez JJ. *Zea mays* subsp. *mexicana* (Mesa-Central subpopulation). The IUCN Red List of Threatened Species 2019. 2019; e.T111341688A111341693. https://dx.doi.org/10.2305/IUCN.UK.2019-2.RLTS.T111341688A111341693.en. Accessed on 20 July 2022.
7. de la Cruz Larios L, Ruíz Corral JA, Aragón Cuevas F, González Ledesma M, Contreras A, Oliveros O, Sánchez JJ. *Zea mays* subsp. *mexicana* (Nobogame subpopulation). The IUCN Red List of Threatened Species 2019. 2019; e.T111341662A111341680. https://dx.doi.org/10.2305/IUCN.UK.2019-2.RLTS.T111341662A111341680.en. Accessed on 20 July 2022.
8. Contreras A, de la Cruz Larios L, González Ledesma M, Ruíz Corral JA, Aragón Cuevas F, Sánchez JJ. *Zea mays* subsp. *parviglumis*. The IUCN Red List of Threatened Species 2019. 2019; e.T107674570A107674577. https://dx.doi.org/10.2305/IUCN.UK.2019-2.RLTS.T107674570A107674577.en. Accessed on 20 July 2022.
9. Sánchez JJ, Ruíz Corral JA, de la Cruz Larios L, Contreras A. *Zea perennis*. The IUCN Red List of Threatened Species 2019. 2019; e.T77727073A77727085. https://dx.doi.org/10.2305/IUCN.UK.2019-2.RLTS.T77727073A77727085.en. Accessed on 20 July 2022.
10. NOM-059-SEMARNAT-2010, Protección ambiental-Especies nativas de México de flora y fauna silvestres-Categorías de riesgo y especificaciones para su inclusión, exclusión o cambio-Lista de especies en riesgo. 2010
